# A Shape Memory Alloy-Based Soft Actuator Mimicking an Elephant’s Trunk

**DOI:** 10.3390/polym15051126

**Published:** 2023-02-23

**Authors:** Minchae Kang, Ye-Ji Han, Min-Woo Han

**Affiliations:** 1 Advanced Manufacturing & Soft Robotics Laboratory, Department of Mechanical Engineering, Dongguk University, 30 Pildong-ro 1, Jung-gu, Seoul 04620, Republic of Korea; 2 Advanced Manufacturing & Soft Robotics Laboratory, Department of Mechanical, Robotics and Energy Engineering, Dongguk University, 30 Pildong-ro 1, Jung-gu, Seoul 04620, Republic of Korea

**Keywords:** actuator, elephant trunk, shape memory alloy, manipulator, nature-inspired, artificial muscle

## Abstract

Soft actuators that execute diverse motions have recently been proposed to improve the usability of soft robots. Nature-inspired actuators, in particular, are emerging as a means of accomplishing efficient motions based on the flexibility of natural creatures. In this research, we present an actuator capable of executing multi-degree-of-freedom motions that mimics the movement of an elephant’s trunk. Shape memory alloys (SMAs) that actively react to external stimuli were integrated into actuators constructed of soft polymers to imitate the flexible body and muscles of an elephant’s trunk. The amount of electrical current provided to each SMA was adjusted for each channel to achieve the curving motion of the elephant’s trunk, and the deformation characteristics were observed by varying the quantity of current supplied to each SMA. It was feasible to stably lift and lower a cup filled with water by using the operation of wrapping and lifting objects, as well as effectively performing the lifting task of surrounding household items of varying weights and forms. The designed actuator is a soft gripper that incorporates a flexible polymer and an SMA to imitate the flexible and efficient gripping action of an elephant trunk, and its fundamental technology is expected to be used as a safety-enhancing gripper that requires environmental adaptation.

## 1. Introduction

The bio-simulating actuator based on soft materials can offer a wide variety of possible applications, including those requiring adaptability. Accordingly, studies on soft actuators imitating animals are actively being conducted to attain their own aim [1,2,3]. These soft actuators are usually inspired by the muscles of an organism [4,5]. In particular, since it is generally made of a polymer, it has advantages such as continuity and infinite degrees of freedom (DOF). Since the polymer is soft, it demonstrates a high level of safety, and can be utilized as a soft actuator to cope with difficult environments [6,7,8]. However, these actuators have disadvantages such as a low loading capacity. In addition, the location detection and path planning are not common, making them difficult to control.

An elephant’s trunk shows flexible movements that are difficult for machines to imitate. This is because the trunk is made up of several muscles without bones. The belly side of the trunk has fine wrinkles, making it easy to lift the object without slipping when holding it [9]. By emulating this with a soft material, an actuator that mimics an elephant’s trunk may have lower occupational risk and excellent environmental adaptability.

Elephant trunks are exclusively composed of boneless muscles, which embody various movements [10]. However, in most papers, the elephant-simulating actuator was constructed using mechanical joints to implement these elephant trunks [11,12]. Using mechanical joints and various numbers of motors makes path setting complicated and difficult to make a continuous motion. In addition, damage may occur in relation to force control during movement.

Thermally responsive shape memory alloys (SMAs) play the role of muscles in soft actuators [13,14,15]. SMAs can find the original shape with a phase transformation. This phase change occurs in SMA according to temperature variations [16,17]. This phase transition between the low-temperature martensite and the high-temperature austenite creates motion and force. Therefore, by using SMAs together with soft actuators, they perform various movements without using a motor. Furthermore, the complexity of the actuator may be decreased because it not only occupies less space than the motor but also simply creates flexible movements using wire-type actuating sources.

Furthermore, the SMA springs used in the soft actuators a have higher energy density, strain, and flexibility than the SMA wire, making it the basis for various biomimicry robots [18,19,20]. These soft robots have the advantage of being able to achieve various environmental adaptations through multi-motion using SMA springs. A robot that crawls like an earthworm has been presented. This robot was built by utilizing the SMA spring-based actuator for the flexible movement of earthworms [21]. The robot, which simulates a turtle, implements bending motions through the SMA springs’ on–off system. This also allows crawling on the floor without precise control [22]. In addition, the octopus-mimicking robot showed an advanced performance using SMA springs and the proportional–integral (PI) controller to imitate the octopus [23].

Several 3D printing procedures have been used to combine the SMA with the soft polymer. With 3D printing, a soft polymer that takes a long time to cure can be finalized into the desired shape. SMA actuators were integrated into plastic 3D-printed structures that contained two 3D-printed sensors to create the soft finger [24]. Additive manufacturing-based bioinspired structures capable of combining materials with various stiffness have been created. It is a smart structure made of flexible and rigid components by activating the SMA [25]. Furthermore, a custom-made 3D printing system was developed to embed the SMA wires into the soft material. This makes it possible to create complex shapes for lightweight, adaptable aerospace components [26].

In order to create intricate shapes, 3D printing is also useful. A 3D-printed core and mold were used to create a three-finger gripper attached to the robot arm. Silicon is placed into a core that is fixed to a 3D-printed mold [27]. Moreover, complicated air channels were successfully created inside flexible polymers utilizing molds manufactured by the 3D printing process [28].

As part of our research, we developed a soft actuator that mimics the motion of an elephant’s trunk. The body of the actuator is constructed from a soft polymer, and the integrated SMA springs respond to heat so as to provide flexible and diverse motions of the elephant trunk. Each SMA spring is independently controlled by a current channel to achieve a phase change in the SMA springs positioned in each of the soft actuator’s four quadrants. The phase transformation of the embedded SMA springs was induced by ohmic heating, making it feasible to create bending and twisting movements. To investigate the actuation performance, the amount of deformation in response to the application of a current was measured, and a cup filled with water was lifted to simulate a load test. Lastly, in order to evaluate the performance of the actuator, an experiment was conducted to lift various types of objects.

## 2. Materials and Methods

### 2.1. Biomimicry of An Elephant’s Trunk

**Figure 1:**
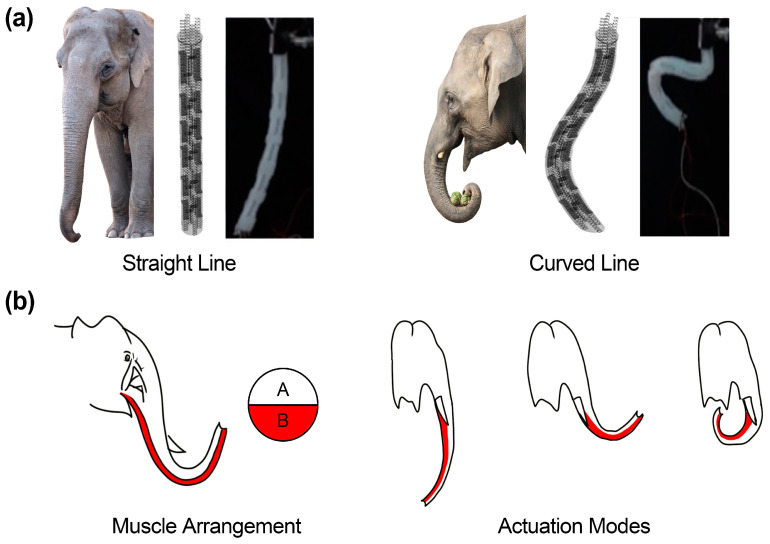
(**a**) Images of the elephant trunk, the 3D model of the elephant trunk actuator; and the actual actuator; (**b**) Muscles of an elephant’s trunk; elephant trunks are composed of two long muscles, which can produce various movements, redrawn from [29].

Elephant trunks are composed of muscles, blood vessels, nerves, fat, other connective tissue, and skin, without bones and cartilage. First of all, their various movements and flexibility are enabled by a large number of muscles, which are approximately 150,000 pieces. The superficial muscles extend vertically with the back, abdomen, and side of the trunk. It was confirmed that these elephant muscles are arranged in a straight line on the pillar [9] and were manufactured in a similar form in the actuator to reproduce them. Figure 1a shows the elephant trunk and the proposed actuator. The stretched form of the elephant’s trunk represents the state before actuation, while the curled shape of the elephant’s trunk is realized by actuated condition. The elephant’s trunk predominantly consists of lengthy muscles in its upper and lower regions. According to the contraction and tension of the muscles, it is possible to express up and down bending motions, as seen in Figure 1b.

### 2.2. Materials, Fabrications, and Evaluation Methods

To simulate this elephant trunk, soft silicone (Dragon Skin 30; Smooth-On, Inc., Macungie, PA, USA), 3D-printed molds, and SMA springs were used. Dragon skin 30 has high elasticity and high strength, so it returns to its original state even if it is forced and deformed. Because of these characteristics, it has recently been used to manufacture various soft robots and actuators [8,30]. In this research, these properties also enabled the implementation of repeating motions overcoming the shrinkage of the polymeric matrix due to the embedded SMA springs. The material properties of dragon skin 30 are shown in Table 1.

**Figure 2:**
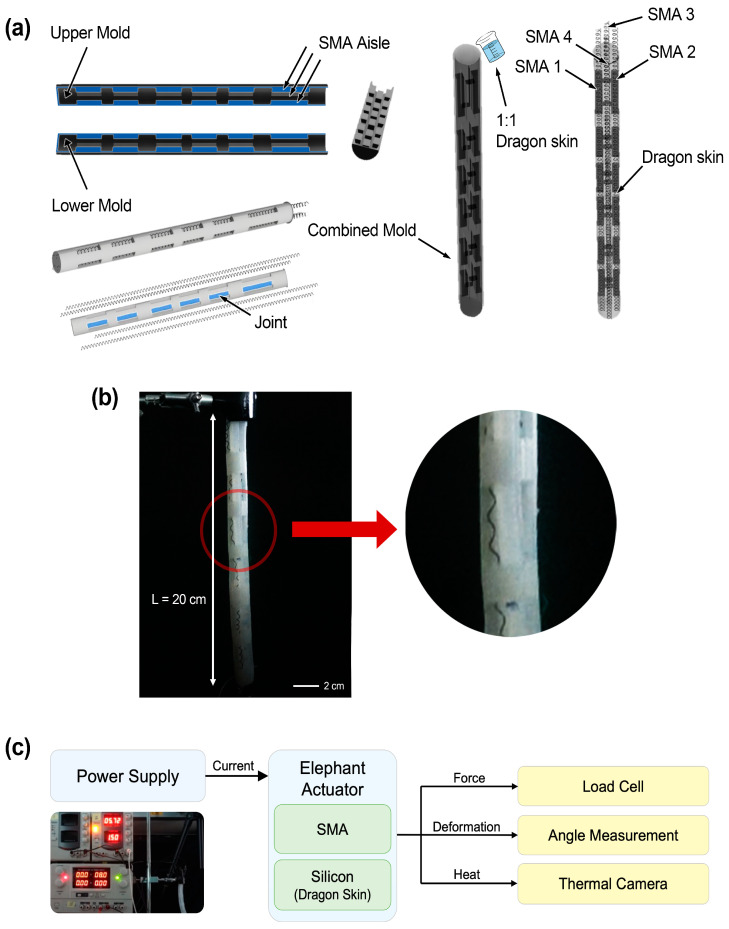
(**a**) Fabrication of an elephant trunk actuators. (**b**) Shape of an elephant trunk actuator. (**c**) Evaluation procedures of the actuators; the force was measured concerning the movement shown when the current was applied to the actuator. The displacement was checked through the angle, and it was also confirmed that the heat was observed through the thermal imaging camera.

To obtain the solid-types of desired shape, the liquid polymer was casted. In the casting procedure, 3D-printed molds were used. In order to insert four SMA springs, a long cylindrical mold was divided into two parts as shown in Figure 2a. The mold consists of upper and lower parts, and the route for the SMA springs is a channel through which long squares are organized side by side. After the SMA spring was inserted, the two halves of the mold are combined into one part. Then, the whole produced component is put in a single mold, and the additional polymer is poured onto it (Figure 2a). The shape of the completed actuator is shown in Figure 2b, and the total length (L) is 20 cm. For the manufactured actuator, an experiment was conducted to confirm its performance by applying a current using a power supply as specified in Figure 2c. The force was measured using a load cell, and the deformation was confirmed through an angle measurement, and finally, it was taken with a thermal imaging camera.

## 3. Results

### 3.1. Experiments on SMA Springs

**Figure 3:**
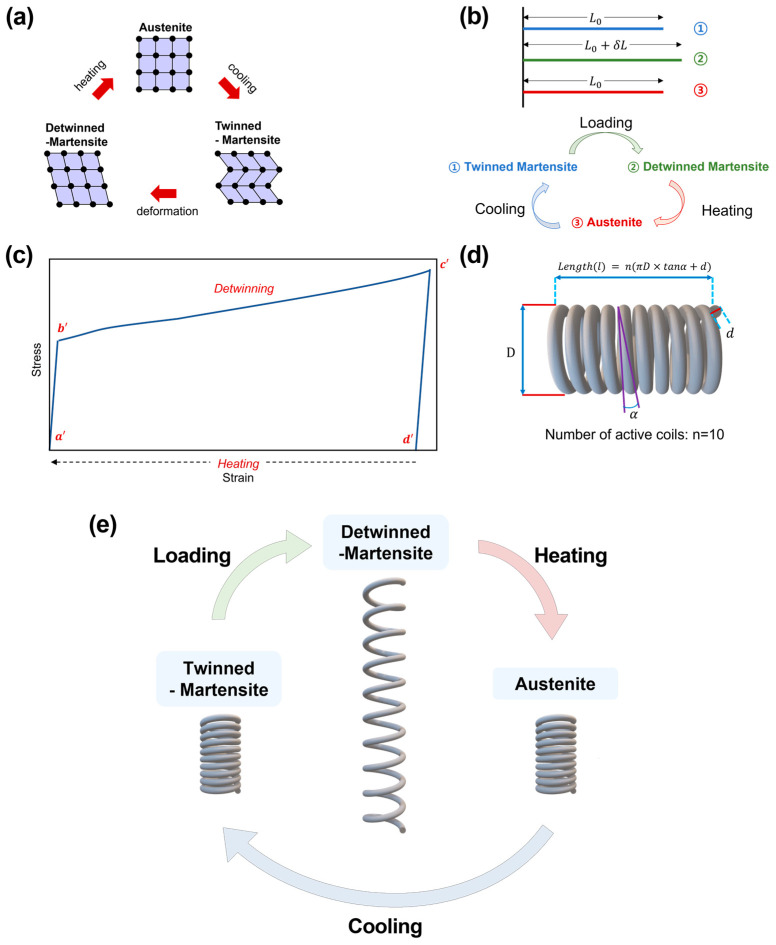
(**a**) Phase transformation of SMA. (**b**) The length changes according to the phase change of SMA. (**c**) Stress–strain graph when the phase change of SMA occurs. (**d**) Variables of SMA spring. (**e**) Phase transformation of the SMA spring corresponding to length change [32,41,42].

If the temperature of the SMA is higher than the transformation temperature, it will be changed to the stored shape, as shown in Figure 3a. There are several types of SMA [32]. The SMA used here is based on NiTi, which is typically made of 55–56% nickel and 44–45% titanium and initiates the advantage of having flexibility against wear resistance [33,34]. Detailed material properties are shown in Table 2 [35,36,37,38,39,40]. The thermoelastic phase transformation alters the geometry of the SMA, as seen in Figure 3b. In its martensitic phase, the SMA is deformable and recovers its original shape when heated (shape memory effect, SME). Changes in the axial length of up to 8% for SMA wire and 22.5% for SMA springs are especially possible [34]. Detwinning is a unique feature of SMA, where the material is deformed beyond the elastic limit in the martensite phase. Figure 3c shows an SME stress–strain graph [41]. If the load exceeds the point of *c*’ in Figure 3c, it cannot regain its original shape and is irreversibly deformed or damaged. If loading stopping before that, then *c*’ linearly restores the strain to point *d*’. The residual strain during the path *d*’–*a*’ can be recovered by applying heat. After this step, cooling allows the material to reach its initial state.

The SMA spring is shown in Figure 3d with its variables. Figure 3e shows that the SMA spring is also changed with temperature. When an austenitic spring is coiled, it transforms into a twinned martensite and is ready to begin a new actuation cycle. When an external force stretches the spring, the twinned martensite phase becomes the detwinned martensite phase. The deformed spring will return to a memorized contracted shape at temperatures above the austenite starting temperature. The SMA spring is repeatedly actuated in this manner.

**Figure 4:**
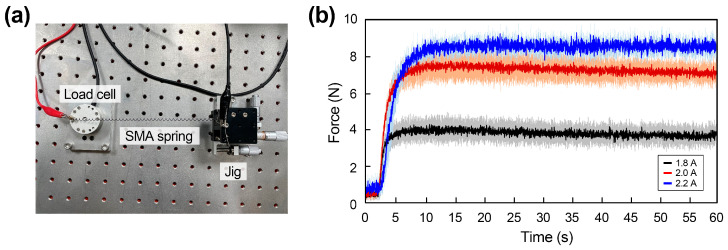
(**a**) Experiment setup for the force measurement of the SMA spring; and (**b**) Force–time relationship graph of one SMA spring with the increasing current.

An experiment was conducted on the SMA spring, and the setup was shown in Figure 4a. When the 1.8 A, 2.0 A, and 2.2 A of current were applied to the SMA spring, the force was measured using a force torque sensor. As shown in the results in Figure 4b and Table 3, the force increases when a high current value is applied. The maximum value for 1.8 A is 4.3 N, whereas the maximum value for 2.0 A is 7.8 N, and the maximum value for 2.2 A is 9.3 N.

### 3.2. Performance Evaluations of Bending Behaviors of Elephant Trunk Actuators

#### 3.2.1. Effects of the Number of Soft Joints in the Actuators

**Figure 5:**
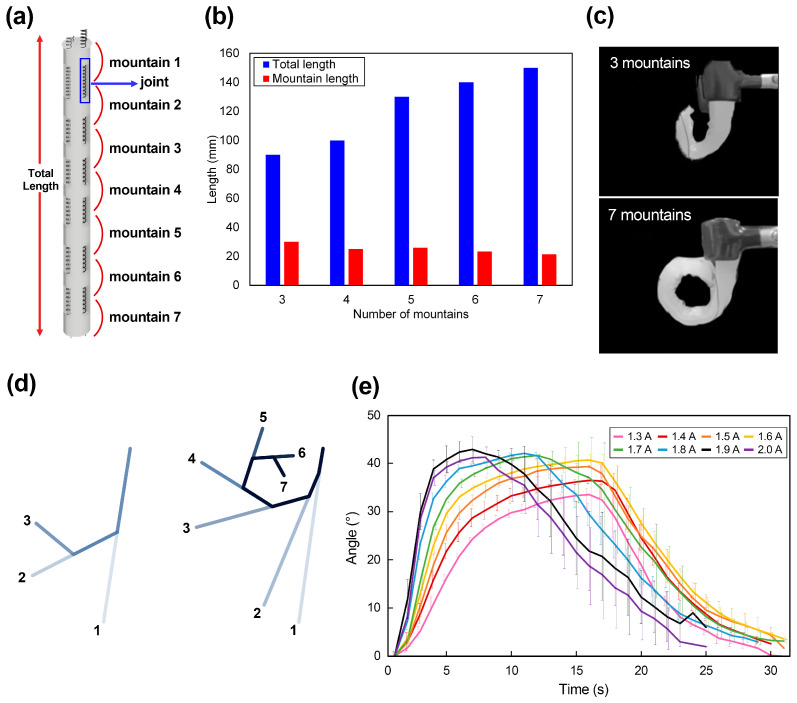
(**a**) Conceptual view of the soft actuator; (**b**) The total length of each actuator according to the number of mountains; (**c**) Images of deformed actuators—these show the shape when there are three and seven mountains; (**d**) Deformation results depending on the number of mountains. The numbers in the figure indicate the number of mountains; and (**e**) Deformation angle with different input currents. In the test, the actuator with seven mountains was the test subject.

The goal of the following experiments was to observe the deformation according to the number of soft joints in the elephant trunk actuator. As shown in Figure 5a, a soft joint is a part of the actuator where the SMA spring is exposed from the polymer body. When the number of joints is *n*, the number of created mountains is *n + 1*. The term “*mountain*” refers to a shape in which a polymer rises due to bending. Since the lengths of the soft joints are equal for all actuators, the total length of an actuator depends on the number of joints.

In addition, it was determined that the length of each mountain formed by bending is distinct for each actuator fabricated with a different number of joints (Figure 5b). According to Figure 5b, as the number of mountains increases, the length of each mountain decreases. Figure 5c,d show the actuators’ deformation with three and seven mountains. The actuator with three mountains connected by two joints has a maximum deformed shape similar to the character “*U*”, while the actuator with seven mountains connected by seven joints deforms to make a shape similar to the character “*O*”. It was observed that, as the number of mountains increased, the deformed shape eventually became more circular.

#### 3.2.2. Relationships between Electric Current and Deformation

Depending on the magnitude of the current applied to the SMA spring, the maximum deformation of the actuator and the time required to achieve maximum deformation could be varied. In order to quantify this and use it for multiple actuations, the current was applied to the soft actuator in units of 0.1 A from 1.3 A to 2 A. The current was cut off when the actuator was no longer deformed. In addition, the whole period between when the actuator began to deform due to the application of the current and when it returned to its original condition was recorded. The average value for deformation was obtained after more than three rounds of experiments for each current, and the results are shown in Figure 5e. In the test, the actuator with seven mountains was evaluated. The heat of the resistance generated through the current made a significant difference in actuating conditions. Additionally, the elastic restoring force of the polymer matrix enables this soft actuator to smoothly return to its original form when we cut off a power source.

On a 2.0 A case, it took 7 s to achieve the maximum angle (43°) and 19 s to return to the initial position. As a more exact experimental result, it does not completely return to its original location but averages a 96.8% recovery rate. It was confirmed that, as the current increases, the change in angle tends to appear larger in a relatively shorter time. When currents of 1.3 A and 2.0 A were applied to the actuators, their maximum angle was 34° and 43°, so there was a difference of approximately 10 degrees when comparing the maximum reaching angle. From 1.6 A, there was a difference in the time to reach the maximum angle as the applied current value increased. However, there was no significant difference in the maximum angle generated. On the other hand, the higher the current is, the longer it takes to restore. At 1.3 A, it took 13 s to return to its original position at the maximum angle, but at 2.0 A, it took 19 s. This is because the difference in the maximum angle and the high resistance heat generated according to the high current were maintained. In this experiment, restoration after cutting off the current at a room temperature of 23 degrees was confirmed without using a special cooling system. The reason why it was able to return to its original position is because of the elasticity of Dragon Skin 30 and gravity due to the original weight of the actuator.

In this study, a separate cooling system was not used to restore the actuator to its original position after operating it, but the performance can be further improved using the cooling method. Other previous studies using SMA have used the following methods. A multi-input–multi-output controller was proposed to enable active cooling with compressed air. Through this controller, the SMA quickly switched SMA’s heating and cooling state, which implements the tentacle’s bending posture in three-dimensional (3D) space [43]. An SMA spring actuator with a cooling function was presented. In order to facilitate the real-time control of the SMA spring, a new operating mechanism is proposed to release the air out of the water using water as a cooling medium. This resulted in higher robot speeds [44]. A soft three-modular-section robot uses an SMA spring as an actuator. For fast actuation, three air tubes are integrated and provide a forced convection function that shortens the cooling time of the SMA spring [45]. In future work, it will be possible to apply this to develop an actuator that shows motion by faster driving.

### 3.3. Evaluations for Multi-Axis Actuation

#### 3.3.1. Motion Evaluations for Three-Dimensional Deformation

**Figure 6:**
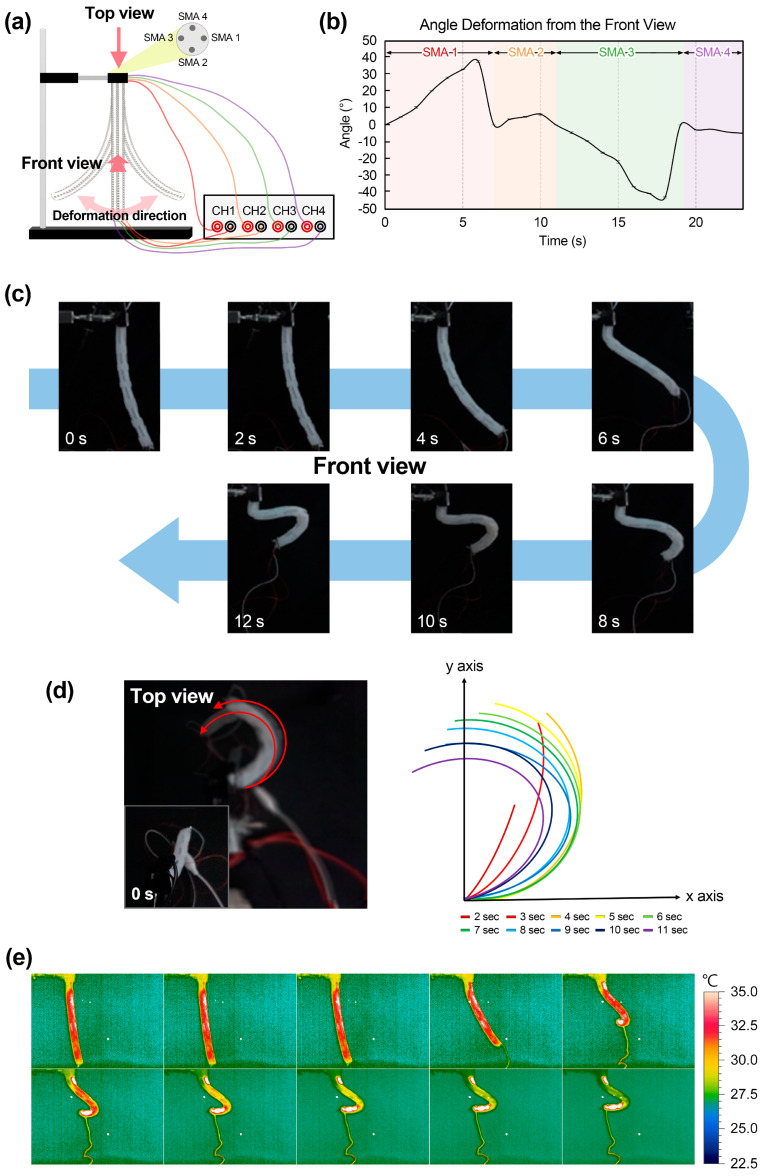
(**a**) Actuation setup for the four-axis elephant trunk actuator. (**b**) Deformation from the front view of the actuator. (**c**) Deformation sequence observed from front view. (**d**) Deformation of the actuator viewed from the top at every two seconds. (**e**) Temperature changes according to current application.

The experimental setting to simulate the movement of the elephant trunk is shown in Figure 6a. The upper part of the actuator is secured to the experimental clamp, and current is applied to the four SMA springs inserted into the actuator. At this time, when the SMA spring was numbered from 1 to 4, the elephant trunk movement was implemented by applying current in order. Figure 6b is a graph showing the angular displacement viewed from the front for the movement generated by the elephant trunk actuator. Figure 6c is a front view photo aligned every two seconds, and the character *S*-shape was slowly generated. Figure 6d shows a top view of an elephant trunk actuator that creates three-dimensional motion as well as the sequence of its deformation. This displacement graph shows the position of the actuator every two seconds, and it was confirmed that the deformed shape of the actuator gradually becomes a circle. These top and front views of movement are shown in Video S1.

In addition, the temperature change that occurs when the current is applied to the actuator was confirmed. The phase transition of the SMA spring is induced by resistance heat resulting from the current passing through it. Figure 6e shows the deformation of the actuator and the associated change in surface temperature when the current is applied. The temperature change identified by the thermal imaging camera started at approximately 22 degrees and increased to 33 degrees. These thermal changes are shown in Video S2.

#### 3.3.2. Lifting Experiments

**Figure 7:**
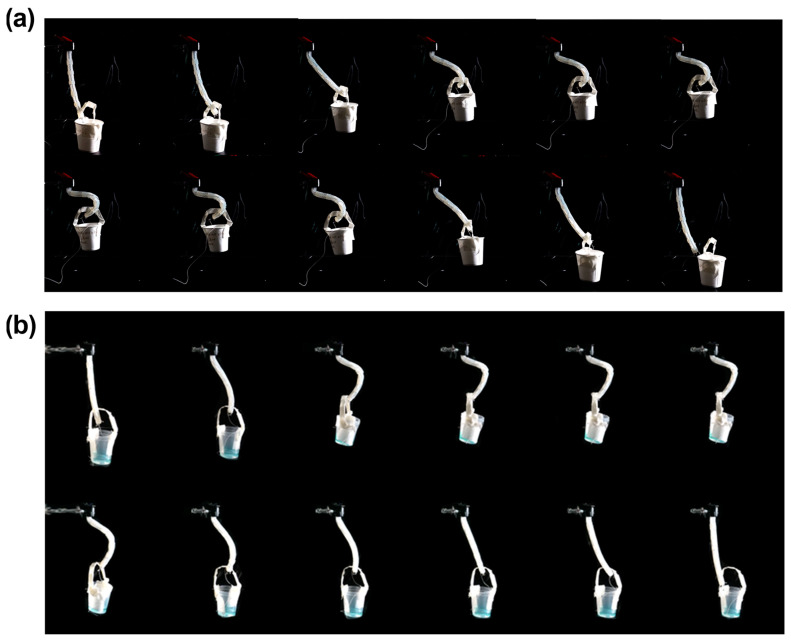
Lifting experiments: (**a**) Soft actuator when lifting and putting down the paper cup; and (**b**) Stably lifting and putting down a cup containing water.

Then, the motion of lifting the object was evaluated. We looked at the motion of the actuator lifting a paper cup with a handle. Figure 7a captures the actuator moving the handle-attached paper cup up every two seconds. Through this picture, it was confirmed that the cup was lifted and landed stably. Following this, an experiment was conducted to determine whether the cups containing water could also be safely transported, as shown in Figure 7b. In the test, we found that the beaker containing 30 mL of water could be safely lifted and landed in its original position. At this time, if you look at the taken images every two seconds, you can observe the blue water fluctuating. This is also shown in Video S3.

**Figure 8:**
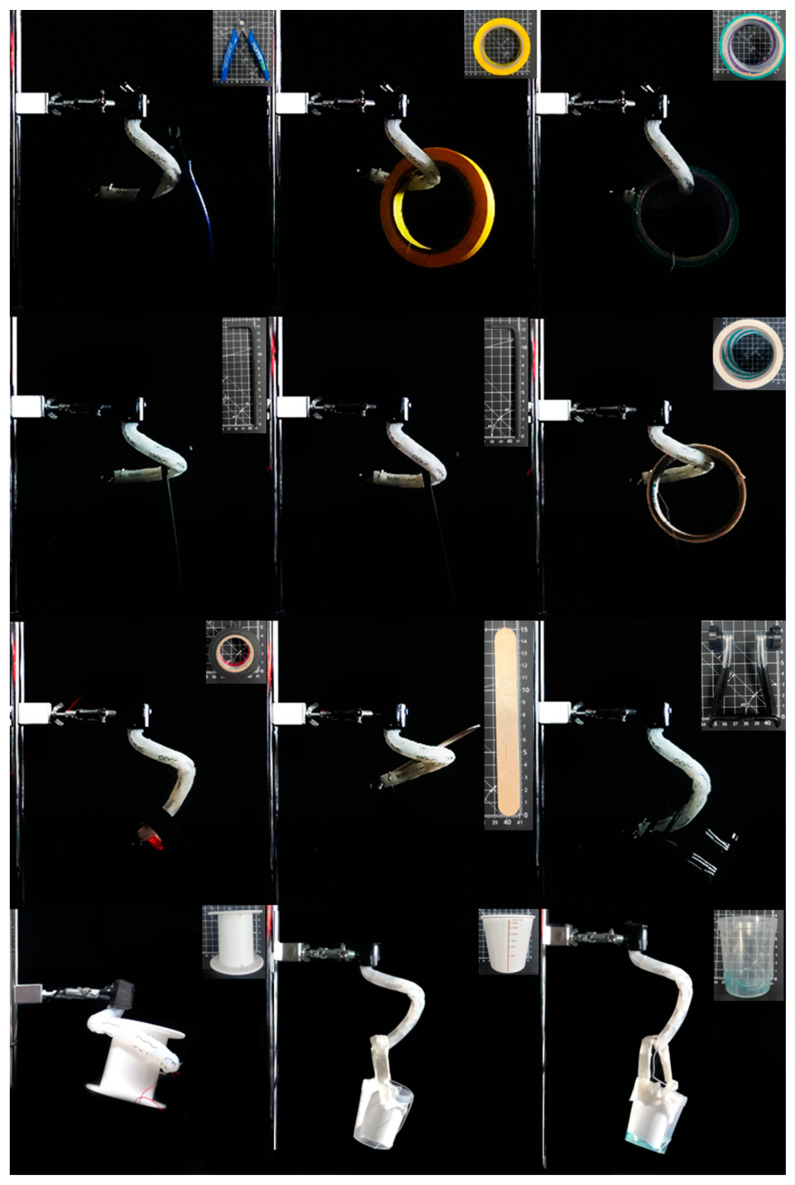
Evaluations of grasping performance: the elephant trunk actuator can grip various objects including yellow paper tape, duct tape, white paper tape, friction tape, a wooden stick, a big hexagonal wrench, a small hexagonal wrench, a phone holder, a nipper, wire container, a paper cup, and a beaker. The target objects were arranged from left to right and from top to bottom.

Finally, experiments were conducted to lift household items of various shapes. The experiment subjects were commercially available items such as tapes, tools, cups, etc. The objects’ lengths varied in shape on a scale by approximately one centimeter, and their weights ranged from 2.3 g to 101.8 g (Figure 8 and Table 4). This actuator, which mimics the form of an elephant’s trunk when it grasps an item, was capable of raising and encircling the objects. Particularly, the actuator performed more effectively in a design where the wrapping motion was favorable. For example, it was capable of lifting a circular tape with a hole in the center, a cup with a handle, and an item with a bent or undercut appearance.

## 4. Conclusions

In this research, a soft actuator that mimics the features of an elephant’s trunk was developed. The actuator imitating an elephant’s trunk was made up of an SMA spring embedded into a soft polymer body, with the SMA spring functioning as a muscle-like actuating source. The phase transformation of the SMA was generated by adjusting the current supplied to it, and the deformation of the whole actuator was created using the contraction–tension behavior that occurs during phase transformation.

To determine whether the form parameters of the developed actuator impact its behavior, soft joints were introduced to the soft actuator, and the behavior patterns were observed in relation to the number of these joints. To further evaluate the actuating characteristics of the actuator according to the current applications, the amount of deformation, deformation rate, and restoration rate produced by altering the amount of current application was observed.

In addition, the elephant trunk actuator was activated by four SMA springs, each of which is independently controlled to produce a variety of movements. In order to use these properties, it was determined that it might be used as a gripper capable of enveloping or lifting an object. To evaluate the grasping performance, the operation of lifting and stably putting down a water-filled cup was successfully completed, as were the verification experiments on the lifting performance of nearby living items of varying shapes and weights.

As a means of improving the utility of the suggested actuator, it may be utilized not only as an independent gripper as in this experiment, but also as a combined end effector with other kinds of manipulators. Above all, in order to enhance the performance of the proposed actuator, it is essential to implement a strategy for precisely controlling the phase transformation of the SMA spring, with an improvement in the gripper design for better grasping performance.

## Figures and Tables

**Table 1:** Material properties of Dragon Skin 30 [31].

Properties	Dragon Skin
Specific gravity	1.08 g/cc
Tensile strength	500 psi
100% modulus	86 psi
Specific gravity	<0.01 in./in.
Shore hardness	30 A
Color	Translucent
Pot life	45 min
Cure time	960 min
Manufacturer	Smooth-On

**Table 2:** Material Properties of SMA.

Parameter	Value	Unit
Martensite start temperature ($M_{s}$)	42	°C
Martensite finish temperature ($M_{f}$)	52	°C
Austenite start temperature ($A_{s}$)	68	°C
Austenite finish temperature ($A_{f}$)	78	°C
Martensite, Young’s modulus ($E_{M}$)	28	GPa
Martensite, Shear modulus ($G_{M}$)	11	GPa
Austenite, Young’s modulus ($E_{A}$)	75	GPa
Austenite, Shear modulus $(G_{A}$)	28	GPa
Diameter of wire	1.37	mm

**Table 3:** Force measurement of the SMA spring by supplying different electric currents.

Duration for Current Supply	1.8 A	2.0 A	2.2 A
10 s	4.0 N	7.4 N	8.3 N
20 s	4.0 N	7.4 N	8.6 N
F_max_	4.3 N	7.8 N	9.3 N

**Table 4:** Properties of the target objects.

Target Objects	Diameter × Height (mm × mm)	Weight (g)	Shape Regularity
Yellow paper tape	108 × 15	57.3	Regular
Duct tape	92 × 15	101.8	Regular
White paper tape	88 × 20	22.8	Regular
Friction tape	53 × 18	35.3	Regular
Wooden stick	150 × 16 × 1	2.3	Slightly irregular
Big hexagonal wrench	145 × 35 × 7	40.6	Highly irregular
Small hexagonal Wrench	125 × 30 × 5	24.6	Highly irregular
Phone holder	60 × 85 × 30	48.8	Highly irregular
Nipper	130 × 90 × 13	58.9	Highly irregular
Wire container	70 × 70 × 65	32.6	Slightly irregular
Paper cup	60 × 70	3.1	Regular
Beaker	60 × 70	10.2	Regular

## Supplementary Materials

The following supporting information can be downloaded at: https://www.mdpi.com/article/10.3390/polym15051126/s1: Video S1: Soft actuator mimicking an elephant trunk; Video S2: Thermal images of soft actuators; Video S3: Lifting test for a water-filled cup.
